# Survival, metabolic activity, and ultrastructural damages of Antarctic black fungus in perchlorates media

**DOI:** 10.3389/fmicb.2022.992077

**Published:** 2022-11-29

**Authors:** Alessia Cassaro, Claudia Pacelli, Silvano Onofri

**Affiliations:** ^1^Department of Ecological and Biological Sciences, University of Tuscia, Largo dell’Università snc, Viterbo, Italy; ^2^Human Spaceflight and Scientific Research Unit, Italian Space Agency, via del Politecnico, Rome, Italy

**Keywords:** perchlorates tolerance, deliquescence, fungal growth, metabolic activity, eukaryotic organism

## Abstract

Evidence from recent Mars landers identified the presence of perchlorates salts at 1 wt % in regolith and their widespread distribution on the Martian surface that has been hypothesized as a critical chemical hazard for putative life forms. However, the hypersaline environment may also potentially preserve life and its biomolecules over geological timescales. The high concentration of natural perchlorates is scarcely reported on Earth. The presence of perchlorates in soil and ice has been recorded in some extreme environments including the McMurdo Dry Valleys in Antarctica, one of the best terrestrial analogues for Mars. In the frame of “Life in space” Italian astrobiology project, the polyextremophilic black fungus *Cryomyces antarcticus*, a eukaryotic test organism isolated from the Antarctic cryptoendolithic communities, has been tested for its resistance, when grown on different hypersaline substrata. In addition, *C. antarcticus* was grown on Martian relevant perchlorate medium (0.4 wt% of Mg(ClO_4_)_2_ and 0.6 wt% of Ca(ClO_4_)_2_) to investigate the possibility for the fungus to survive in Martian environment. Here, the results indicate a good survivability and metabolic activity recovery of the black fungus when grown on four Martian relevant perchlorates. A low percentage of damaged cellular membranes have been found, confirming the ultrastructural investigation.

## Introduction

Since the 1970s, during the NASA Viking lander mission, the presence of oxidizers on Mars surface has been hypothesized from GC–MS data obtained by life detection instruments (Klein, 1978). While the distribution of chlorine was further confirmed by Mars Pathfinder (Gellert et al., 2004), Spirit and Opportunity rovers (Rieder et al., 2004) and Odyssey orbiter (Keller et al., 2006), its chemical form was established only by the NASA Phoenix Mars and Mars Science Laboratory (MSL) landers on Mars (Quinn et al., 2013). The Phoenix’s Wet Chemistry Laboratory analysis found that most of chlorine detected in Martian surface was in the form of perchlorate salts (Hecht et al., 2009; Kounaves et al., 2014), mainly present in regolith as mixture of Ca(ClO_4_)_2_ and Mg(ClO_4_)_2_ with a concentration of 0.4–0.6 wt% and a small amount of Na^+^ and K^+^ (an average of 1.4 and 0.38 mM, respectively; Hecht et al., 2009). Recently, the Sample Analysis at Mars (SAM) instrument on the MSL lander discovered perchlorates in soil samples at Gale Crater (Glavin et al., 2013), the Rosalind Franklin rover landing site. The discovery of perchlorates occurred on Mars, including in Martian meteorite samples (Glavin et al., 2013; Jackson et al., 2015; Jaramillo et al., 2019). The finding of salts in Martian meteorite EETA79001 (the only direct measurement of perchlorates on Martian material) represent an additional confirm of perchlorates presence on the planet (Clark and Kounaves, 2016).

Perchlorates are of great interest to astrobiology and in particular for the habitability of Mars, for their ability to lower the freezing temperature of water (Möhlmann and Thomsen, 2011) and form stable hydrated compounds and liquid solutions by absorbing atmospheric water vapor through deliquescence (Zorzano et al., 2009; Nuding et al., 2014), which allow to create aqueous solution at the appropriate range of temperature (brines) (Martín-Torres et al., 2015). The presence of an aqueous thin film could be important for a putative life-form on Mars, because water is essential for life as we known it (McKay, 2006). However, the presence of perchlorates on Mars surface represents also a challenge for habitability since they can be harmful for life as we know it (Davila et al., 2013; Archer et al., 2015; Wadsworth and Cockell, 2017). These compounds are known as toxic chemicals and strong oxidants (Al Soudi et al., 2017) for cell membranes integrity (Urbansky, 1998), especially when combined with a high radiation environment. Additionally, the presence of salts in a liquid solution may alter the liquid water’s availability, and a low water activity is a basic limit for life because it may result in a desiccation stress (Grant, 2004).

The search for life on Mars is mainly based on the knowledge of surface and subsurface conditions, but also on the survivability of Earth organisms in extreme environments and under Mars-like conditions (Cockell et al., 2016). Mars-like conditions can be found in extremely arid environment like deserts, where the natural occurrence of perchlorates has been generally found (Jackson et al., 2015, 2016). Chlorates and perchlorates highest concentration on Earth has been detected at Lake Vida brines in McMurdo Dry Valleys in Antarctica (Kounaves et al., 2010; Kenig et al., 2016) and in Atacama Desert (Chile) (Trumpolt et al., 2005; Catling et al., 2010), two of the closest terrestrial analogues to Mars past and present environments (Friedmann, 1982; McKay et al., 2003; Onofri et al., 2004; Bishop et al., 2014; Bull et al., 2018; Cassaro et al., 2021).

Several terrestrial microorganisms demonstrate the ability to survive when exposed to perchlorates (Beblo-Vranesevic et al., 2017). Several studies reported that different bacteria strains and only a few archaea are able to metabolize perchlorates, producing chlorite (ClO_2_^−^) as a toxic end-product of (per)chlorate by nitrate reductase (e.g., Coates and Achenbach, 2004; Nerenberg, 2013; Al Soudi et al., 2017; Heinz et al., 2019).

The acidophilic iron sulfur bacterium *Acidithiobacillus ferrooxidans* arrest its growth in the presence of 0.022 M (0.5%) and 0.044 M (1%) of Mg(ClO_4_)_2_ (Bauermeister et al., 2014), while spores of *Bacillus subtilis* are able to germinate up to 0.1 M of NaClO_4_ (Nagler and Moeller, 2015). The bacterium *Hydrogenothermus marinus* showed vitality after an exposure of 5 min in presence of Na-perchlorates (Beblo-Vranesevic et al., 2017).

As reported in Laye and DasSarma (2018), the cold-adapted halophilic Antarctic archaeon *Halorubrum lacusprofundi* demonstrates its ability to grow anaerobically on 0.04 M concentration of perchlorate, while the growth is halved at increased concentrations. *Planococcus halocryophilus* is able to grow in presence of NaCl, MgCl_2_, CaCl_2_, NaClO_4_, Mg(ClO_4_)_2_ and Ca(ClO_4_)_2_, (Heinz et al., 2019). Al Soudi et al. (2017) reported bacterial growth at concentrations of chlorate up to 2.75 M and at 1 M of perchlorate salts. Matsubara et al. (2017) reported the growth ability of halophilic/halotolerant bacterial species (belonging to the genera *Bacillus, Alkalibacillus* and *Halomonas*) from Big Soda Lake (BSL, Nevada). For example, the bacterium *Bacillus licheniformis* survived after the exposure to 5% Na-perchlorate, growth is reduced in the absence of this compound (Matsubara et al., 2017). Recently, Billi et al., 2021 reported a tolerance threshold value of 100 mM perchlorate ions for the two radiation-tolerant cyanobacterium Chroococcidopsis strains and, therefore, suggested their utilization for the *in-situ* resources utilization (ISRU) technologies enabling the future of human space exploration. Also methanogens are able to metabolize concentrations of up to 1% wt/vol of three different perchlorate salt (Na-, Ca- and K-perchlorates) (Kral et al., 2016).

Despite several studies on perchlorate tolerance are reported, the fungal survivability is poorly understood. Fungal survival investigations are mainly focused on chloride species. *Hortaea werneckii* and *Phaeotheca triangularis* grow at up to 25% (w/v) of NaCl, while *Aureobasidium pullulans* grows up to 10% of NaCl (Turk et al., 2004). Recently, Heinz et al. (2021), reported a new record for microbial perchlorate tolerance: the halotolerant yeast *Debaryomyces hansenii* and the filamentous fungus *Purpureocillium lilacinum* (up to 2.4 M and 1.9 M, respectively).

In this context, Life in Space (Origin, presence and persistence of life in space: from molecules to extremophiles), an Italian Space Agency funded project, aims to understand the physiological responses of the cryptoendolithic black fungus *Cryomyces antarcticus* when grown in presence of perchlorate salts. The black fungus, isolated from the Antarctic cryptoendolithic communities, is considered as a eukaryotic test organism in astrobiological studies for its high resistance when exposed, in dehydrated conditions, to full solar radiation (including UV-A, UV-B, and UV-C) (Onofri et al., 2013; Pacelli et al., 2017a); to γ-rays up to 55.81 kGy (Pacelli et al., 2017b), deuterons (up to 1.5 kGy), and to space and Mars-like conditions in Low Earth Orbit (LEO) (Onofri et al., 2012, 2015, 2019); and in hydrated conditions up to 2000 Gy iron ions (Fe^26+^) irradiation (Pacelli et al., 2021) and to high dose of ionizing X-rays (up to 0.3 kGy, Pacelli et al., 2018).

Here, fungal colonies were grown on (i) increasing concentrations of four perchlorate salts to test their tolerance to hypersaline environment and (ii) Mars relevant perchlorate salts (0.4–0.6 wt%) cultivation medium to assess the possibility of survival in a Mars-like environment. Survival, membrane damages and metabolic activity were assessed after 3 months of growth in hypersaline medium.

## Materials and methods

### Test-organism, perchlorates medium preparation, and activity of water measurement

The test organism is the black fungus *C. antarcticus* CCFEE 515, isolated by R. Ocampo-Friedmann from sandstone collected at Linnaeus Terrace in McMurdo Dry Valleys (Southern Victoria Land, Antarctica) by H. Vishniac, during the Antarctic expedition of 1980–1981 (Selbmann et al., 2005). Colonies of the fungus were grown at various concentrations (Table 1) of the following salts: K-perchlorate (KClO_4_), Na-perchlorate (NaClO_4_), Mg-perchlorate (Mg(ClO_4_)_2_), Ca-perchlorate (Ca(ClO_4_)_2_. Each salt was dissolved in distilled water to create perchlorate solutions, which were then filtered using an apparatus with a filtration membrane with 0.2 m pores to reach the same molarity as 1 M. Mars relevant salts medium containing 0.4 wt% of Mg(ClO_4_)_2_ and 0.6 wt% of Ca(ClO_4_)_2_), respectively; was prepared, with a final concentration of 72 mM and 48 mM. All of the salts were first concentrated to a 1 M concentration, and then various dilutions were prepared to get the concentrations listed in Table 1. Due to the limited solubility of the KClO_4_, concentration above 90 mM could not be tested. Culture media were prepared by adding each diluted perchlorate solutions to 2% of Malt Extract Agar (MEA; malt extract, powdered 30 g/l; peptone 5 g/l; agar 15 g/l; Applichem GmbH, Darmstadt, DE). After the solidification of the culture medium on Petri dishes, plates were prepared by plating fungal colonies on each medium. The number of Colony-Forming Units (CFUs) per volume unit of suspension was estimated by using a Bürker chamber (0.100 mm depth, 0.0025 mm^2^): 7 μl from each sample were put into the chamber and the number of CFUs in each of the 4 external 16 group squares was counted. The mean values of CFUs in the four squares were calculated for each count and they were multiplied for the conversion factor 10^4^ in order to estimate the number of CFUs per mL of solution. The colonies were diluted to a final concentration of 20,000 CFU/ml and 0.1 ml (2000 CFUs) from each sample was spread in a Petri dish. Only for Mars relevant substrata, colonies were diluted to a final concentration of 40,000 CFU/ml and 0.1 ml (4,000 CFUs) was plated. Fungal colonies were grown at 15°C that is the optimal grow temperature for 3 months; the experiment was performed in quadruplicates. After 3 months, the ability of the fungal cells to growth on culture medium at different concentrations of perchlorates (Table 1), was investigated by counting the number of grown CFUs, and by comparing them with a control (Ctr, 0 mM) samples (grow on physiological conditions, 0 mM of perchlorates). The activity of water (a_w_) of each medium was measured by using an HygroPalm HP23-AW-A (Rotronic, Bassersdorf, Germany) water activity meter (Decagon Devices, Inc., Pullman, WA). The instrument was run at room temperature, and providing 3 output data: percentage of water present in the plate with the culture medium and colonies, ratio in g/Kg to the amount of water present, ambient temperature.

**Table 1:** Concentrations (*) and %wt for each tested perchlorate.

NaClO_4_	%wt/vol [w/v]	KClO_4_	%wt/vol [w/v]	Mg(ClO_4_)_2_	%wt/vol [w/v]	Ca(ClO_4_)_2_	%wt/vol [w/v]
5	0.06	5	0.07	5	0.16	5	0.12
50	0.61	50	0.7	50	1.6	50	1.19
150	1.84	80	1.11	70	2.31	100	2.39
190	2.32	90	1.25	80	2.65	160	3.8
200	2.44			100	3.31	170	4.06
210	2.57			120	4	180	4.30
220	2.69			140	4.6	190	4.54
230	2.82			145	4.80	200	4.78
				150	5	210	5.02

*Concentrations are expressed in millimolarity (mM).

### Metabolic activity estimation by MTT (3-(4,5-dimethylthiazol-2-yl)-2,5-diphenyltetrazolium) assay

For the metabolic activity analyses, a concentration of 3.5 × 10^5^ cells/mL of fungal colonies grown on different perchlorate concentration culture medium was taken with a sterile loop and added into a suspension containing a solution of 0.5 mg/ml of MTT in Phosphate-Buffered Saline (PBS). 100 μl of the suspension were placed into 96-well microplates. Two separate tests were performed incubating cells for 48 and 72 h, respectively. After incubation at room temperature for 48 and 72 h, MTT solution was removed with a multi-channel pipette and 100 μl of DMSO (DiMethyl SulfOxide) was added on each well. The absorbance of each samples was read at 595 nm with a microplates spectrophotometer (BMG NOVOstar microplate fluorometer, MTX Lab Systems), after time intervals of 10, 20 and 30 min, subtracting the absorbance relative to the wells containing only MTT reagent. Results of the absorption were normalized by the number of cells per well and these values were normalized by dividing by the non-treated samples (Ctr).

### Cell membranes integrity assessment

#### Propidium MonoAzide (PMA) assay

Quantitative PCR (qPCR) with PMA was performed to assess membrane integrity after irradiation exposure. One mille liter of fungal colonies at concentration of 20,000 CFU/ml were divided in two identical aliquots of 500 μl. Only one aliquot was treated with PMA as following: a solution of 5 μl of PMA (20 mM concentration, Biotium, Hayward, CA, United States) was added the samples. Both aliquots (treated with PMA and non-treated) were kept in the dark in a constant-shaking incubator for 1 h. PMA solution penetrates only cells with damaged cell-membranes and crosslinks the DNA preventing PCR after being exposed to light (Onofri et al., 2012). DNA extraction was performed using NucleoSpin® Plant kit (Macherey-Nagel, Düren, Germany) following the protocol optimized for fungi Selbmann et al. (2011). The extracted DNA was quantified using the Qubit dsDNA HS Assay Kit (Thermo Fisher Scientific, Massachusetts, United States) and normalized at the same concentration of 0.1 ng/ml. qPCR assay was performed to quantify the number of fungal Internal Transcribed Spacer (ITS) ribosomal DNA fragments (281 bp) present in treated and non-treated samples. A detailed protocol is provided by Onofri et al. (2012). All tests were performed in triplicate.

### Ultrastructural investigation

#### Transmission Electron Microscope (TEM)

Colonies grown on increased concentration of perchlorate culture medium were prepared for microscopy analyses according to the protocol reported in Pacelli et al. (2017a). TEM observations were performed with a JEOL 1200 EX II electron microscope at the Great Equipment Center, section of Electron of the University of Tuscia (Viterbo, Italy). Micrographs were acquired with an Olympus SIS VELETA CCD camera and iTEM software.

## Results

### Survival ability in perchlorate media

The growth ability of *C. antarcticus* under perchlorate salts was assessed by counting the colony-forming units (CFUs) after 3 months of incubation in perchlorate medium (Table 1).

**Figure 1:**
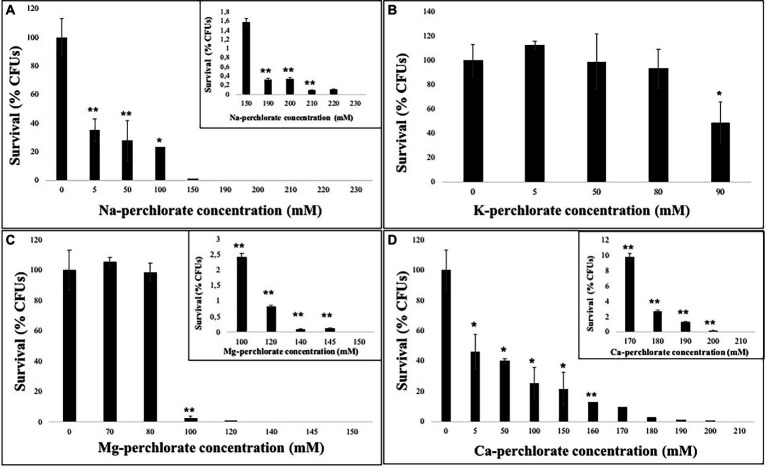
Survival ability of *C. antarcticus* colonies after growth on different perchlorates concentrations. **(A)** Na-, **(B)** K-, **(C)** Mg-, and **(D)** Ca-perchlorates. All concentrations are expressed in mM. Significant differences were calculated by *t test* with * = *p* < 0.05 and ** = *p* < 0.001.

Overall, a common trend in the fungal growth was detected: a decrease as the perchlorate concentration increases. Figure 1A showed a reduction in fungal growth of around 60% already at 5 mM of Na-perchlorate; however, survivors are present even at 220 mM of Na-perchlorate (Figure 1A, inset).

The solubility of the K-perchlorate, limited the survival analysis, however 50% of the fungal colonies are able to grow in 90 mM of K-perchlorate medium (Figure 1B). Similarly an excellent survival (up to 98% of survivors) is reported up to 80 mM of Mg-perchlorate with no statistically significant difference compared to the control (0 mM), while a strong reduction in CFUs number is reported at 100 mM (Figure 1C). Only the 2.5% of survivors are detected at the concentration of 100 mM of Mg-perchlorate (Figure 1C, inset). Colonies grown on Ca-perchlorate medium revealed a halving in survivability even from the lowest dose tested (5 mM, Figure 1D), while a concentration of 170 mM of Ca-perchlorate showed around 10% of survivors (Figure 1D, inset).

### Growth tolerance to Mars-relevant perchlorate salts

The survival ability of *C. antarcticus* in the presence of Mars-relevant perchlorate concentrations was investigated after incubation of fungal colonies in 2.4 mM (0.4 wt% of Mg(ClO_4_)_2_ and 0.6 wt% of Ca(ClO_4_)_2_) cultivation medium. An average of 80% of initial colonies survived (Supplementary Figure S1).

### Water activity measurements

The activity of water is considered as a fundamental parameter for the knowledge about the organization and habitability of Martian brines (Toner and Catling, 2016). The water activity is the equilibrium transience of water vapor over a solution (*f*) relative to the *f* transience of water vapor over pure water (*f*_0_) (*aw* = *f*/*f*_0_). This transition process could be approximated by partial vapor pressures (*p* and *p*_0_) at low pressures. In this condition, *a_w_* may be defined as similar to p/p_0_ ratio, which is equivalent to the equilibrium relative humidity (RH) over a salt solution (RH_eq_ = a_w_). The water activity measurements of our samples are reported in Table 2, and are in the range between 0.92 and 0.94.

**Table 2:** Water activity (*a_w_*) values in perchlorates salt solutions.

NaClO_4_	*a_w_*	KClO_4_	*a_w_*	Mg(ClO_4_)_2_	*a_w_*	Ca(ClO_4_)_2_	*a_w_*	T (°C)	**g/Kg
5	0.928	5	0.934	5	0.936	5	0.932	17.6	24
50	0.929	50	0.932	50	0.934	50	0.933	17.6	24
150	0.929	80	0.93	70	0.934	100	0.934	17.6	24
190	0.933	90	0.927	80	0.937	160	0.932	17.6	24
200	0.933			100	0.94	170	0.925	17.6	24
210	0.932			120	0.94	180	0.931	17.6	24
220	0.933			130	0.94	190	0.934	17.6	24
230	0.935			140	0.937	200	0.932	17.6	24
				145	0.937	210	0.934	17.6	24
				150	0.936			17.6	24

^*^Concentrations are expressed in millimolarity (mM).

^**^g/Kg ratio indicates the amount of water in the sample.

### Assessment of metabolic activity through MTT assay

In this study, the MTT assay was performed in order to detect differences in metabolic activity after the growth of fungal colonies to different perchlorate salts. MTT assay was performed only on samples grown on the lower, the intermediated and the higher perchlorate concentration tested.

During the assay, the produced formazan is a clear indicator of the metabolic activity in a cell. The quantity of produced formazan was detected through spectrophotometric measurements, comparing treated and control cells (0 mM). The metabolic activity was tested after 48 and 72 h of DMSO addition, since no differences were reported among different incubation period, only the results after 72 h of incubation were reported.

**Figure 2:**
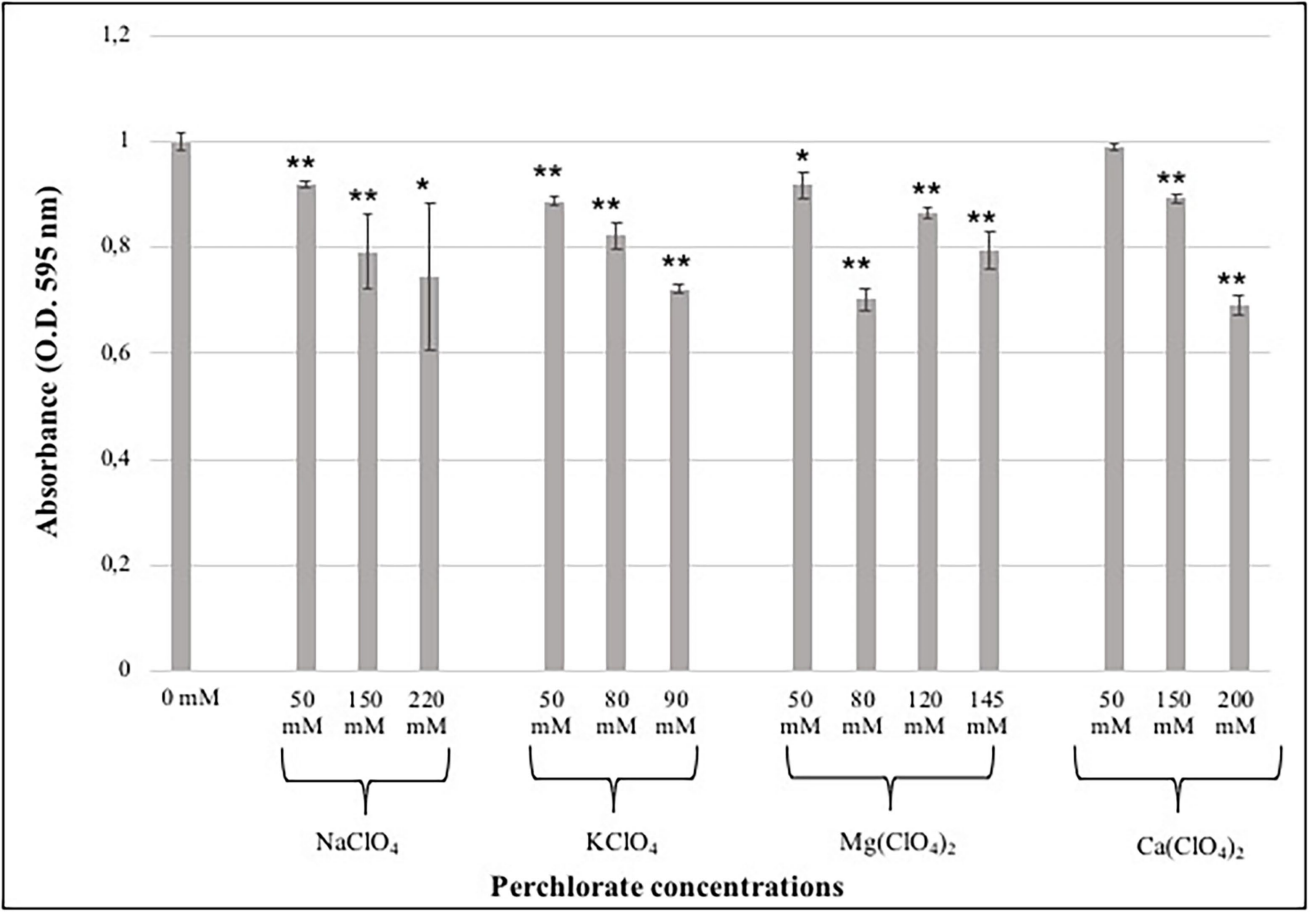
Effect of different perchlorate concentrations on the metabolic activity of *C. antarcticus* colonies. Showed results are after 72 h of incubation with the DMSO. Concentrations are expressed in mM. Data were normalized against the control (0 mM). Significant differences were calculated by *t* test with * = *p* < 0.05 and ** = *p* < 0.001.

The results showed a decrease of metabolic activity as the perchlorate concentration increases, accordingly with survival analyses (Figure 2). Samples grown on Na-perchlorate medium showed a small reduction in metabolic activity already at the concentration of 50 mM, even if around 70% of metabolic activity was maintained at the concentration of 220 mM (Figure 2). A similar trend - decreasing in metabolic activity as perchlorate concentration increases- was observed for samples grown on K- and Mg-perchlorates (Figure 2), with around 70 and 80% of metabolically active cells reported at 90 mM of K-perchlorate and 145 mM of Mg-perchlorate concentrations, respectively. For colonies grown on Ca-perchlorate medium, no statistical differences in metabolic activity were observed between Ctr and 50 mM concentration. A reduction in formazan production, and therefore in metabolic activity, was detected in samples grown on 150- and 200-mM concentrations, with around 90 and 70% of metabolically active cells, respectively (Figure 2).

### Cellular membranes integrity assessment (PMA assay)

**Figure 3:**
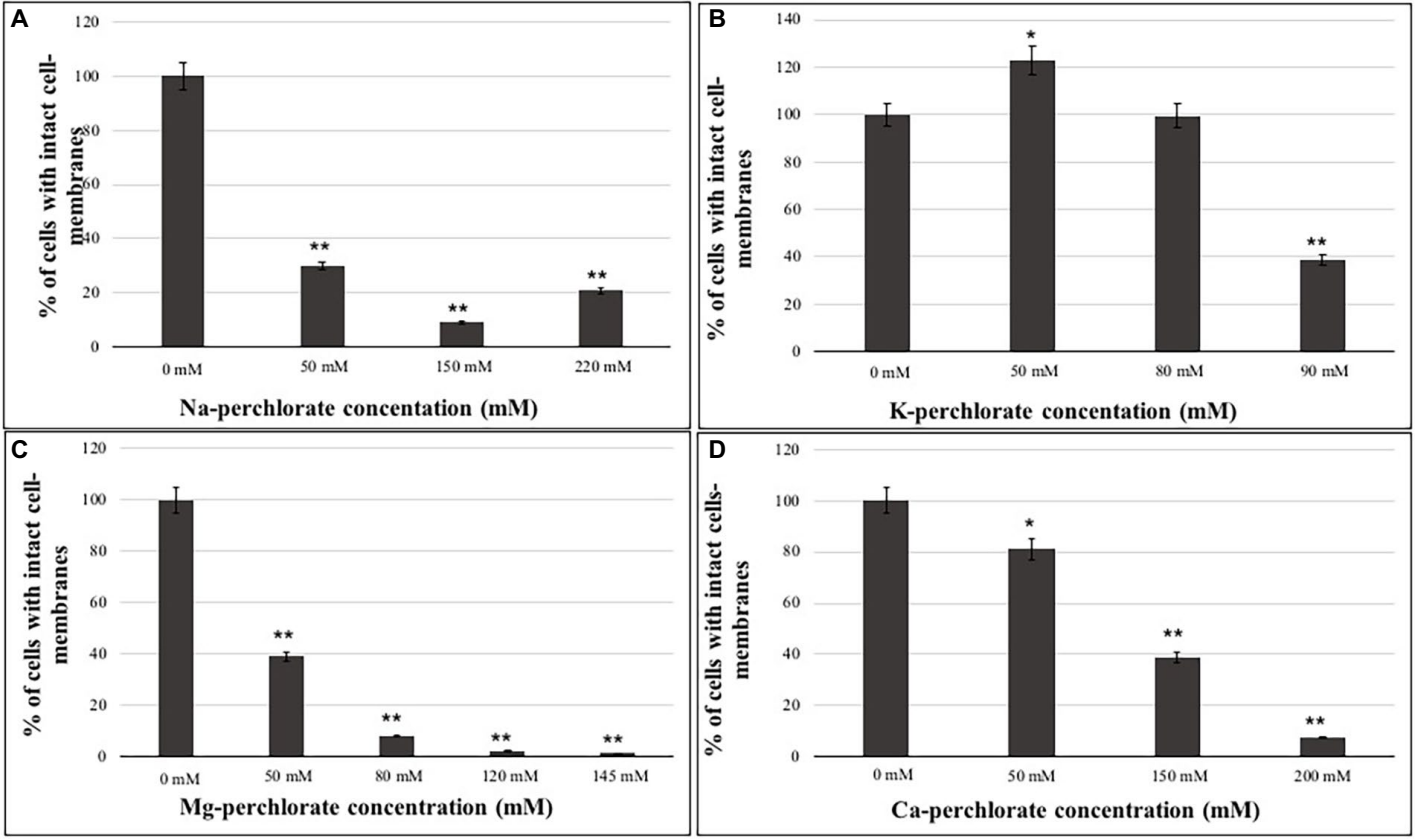
Percentage of cells with intact cell-membranes measured with PMA assay coupled with qPCR of *C. antarcticus* colonies grown on different perchlorates concentrations. **(A)** Na-, **(B)** K-, **(C)** Mg-, and **(D)** Ca-perchlorates. All concentrations are expressed in mM. Significant differences were calculated through *t* test, by comparing 0 mM (Ctr) and the different perchlorate concentrations with * = *p* < 0.05 and ** = *p* < 0.001.

Samples grown at the lower, intermediated and higher perchlorate concentrations were evaluated for their integrity to cell-membranes through PMA assay (Figure 3). As reported for MTT results, PMA results showed a decrease of cells with undamaged cell-membranes as the perchlorate concentration increases (Figure 3). Around 20% of undamaged cell-membranes were detected in samples grown on 220 mM of Na-perchlorate (Figure 3A). Figure 3B shows a good percentage of intact cell-membranes of fungi grown on K-perchlorate up to the concentration of 90 mM (38% of undamaged cell-membranes), confirming of the survival results. Samples grown on Mg- and Ca-perchlorates showed 40% of cells with intact cell-membranes at 50 mM and 150 mM concentrations, respectively (Figures 3C,D). Samples grown on Mg- and Ca-perchlorates showed a strong decrease in the integrity of cell-membranes starting from 80 mM and 200 mM concentrations with only the 8 and 7.5% of survivors, respectively (Figures 3C,D). Values of cells with damaged cell-membranes are also reported in Supplementary material (Supplementary Figure S2).

### Ultrastructural investigation: TEM observations

**Figure 4:**
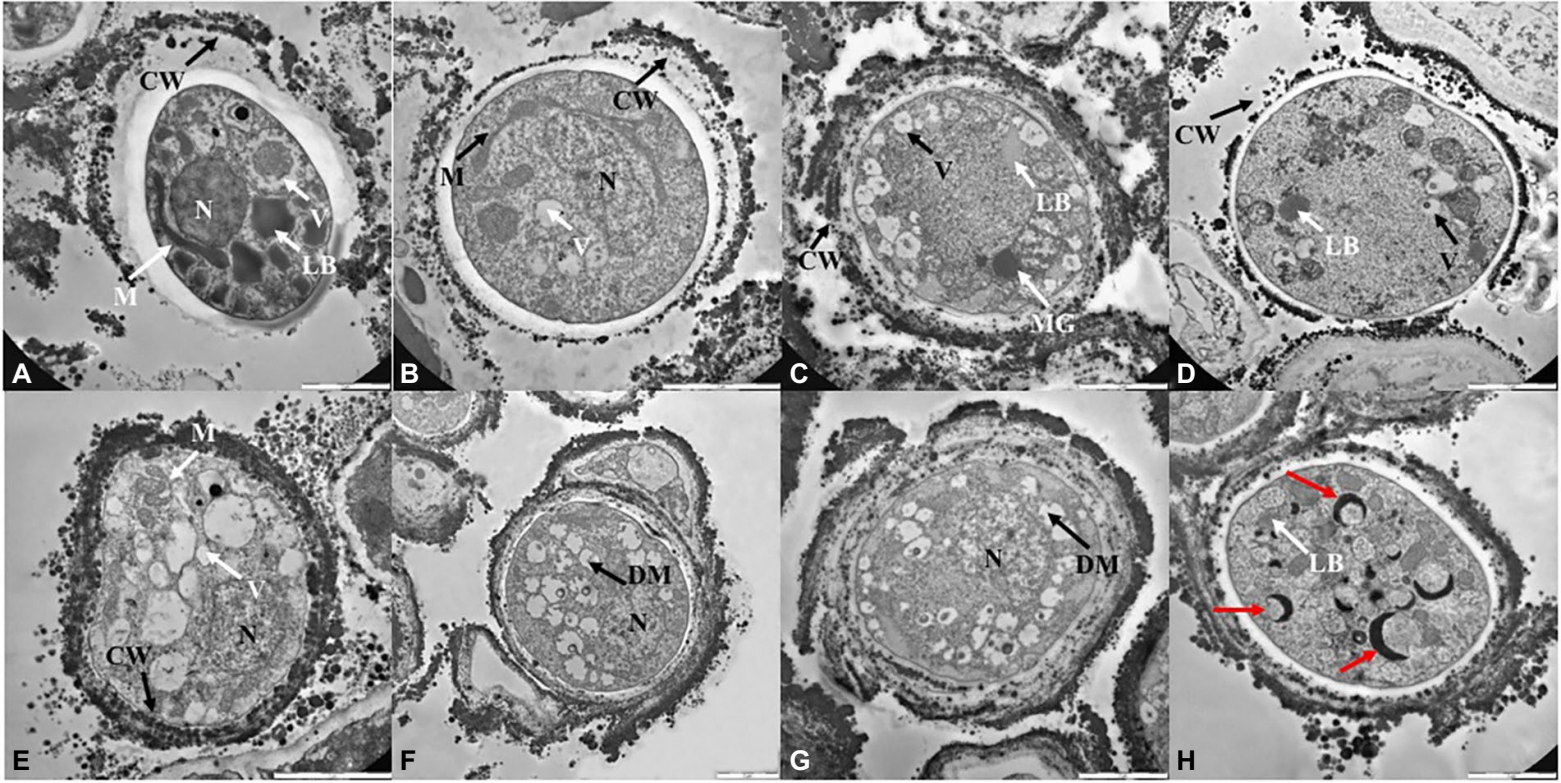
TEM images of *C. antarcticus* cells grown on **(A)** MEA (no salt, control), **(B)** 50 mM, **(C)** 150 mM and **(D)** 220 mM of Na-perchlorate; **(E)** MEA (no salt, control), **(F)** 50 mM, **(G)** 80 mM and **(H)** 90 mM of K-perchlorate. Scale bar: 2 μm. N, nucleus; M, mitochodrion; DM, damaged mitochodrion; CW, cell wall; LB, lipid body; V, vacuoles; MG, melanin granules; red arrow, cell wall indentation.

**Figure 5:**
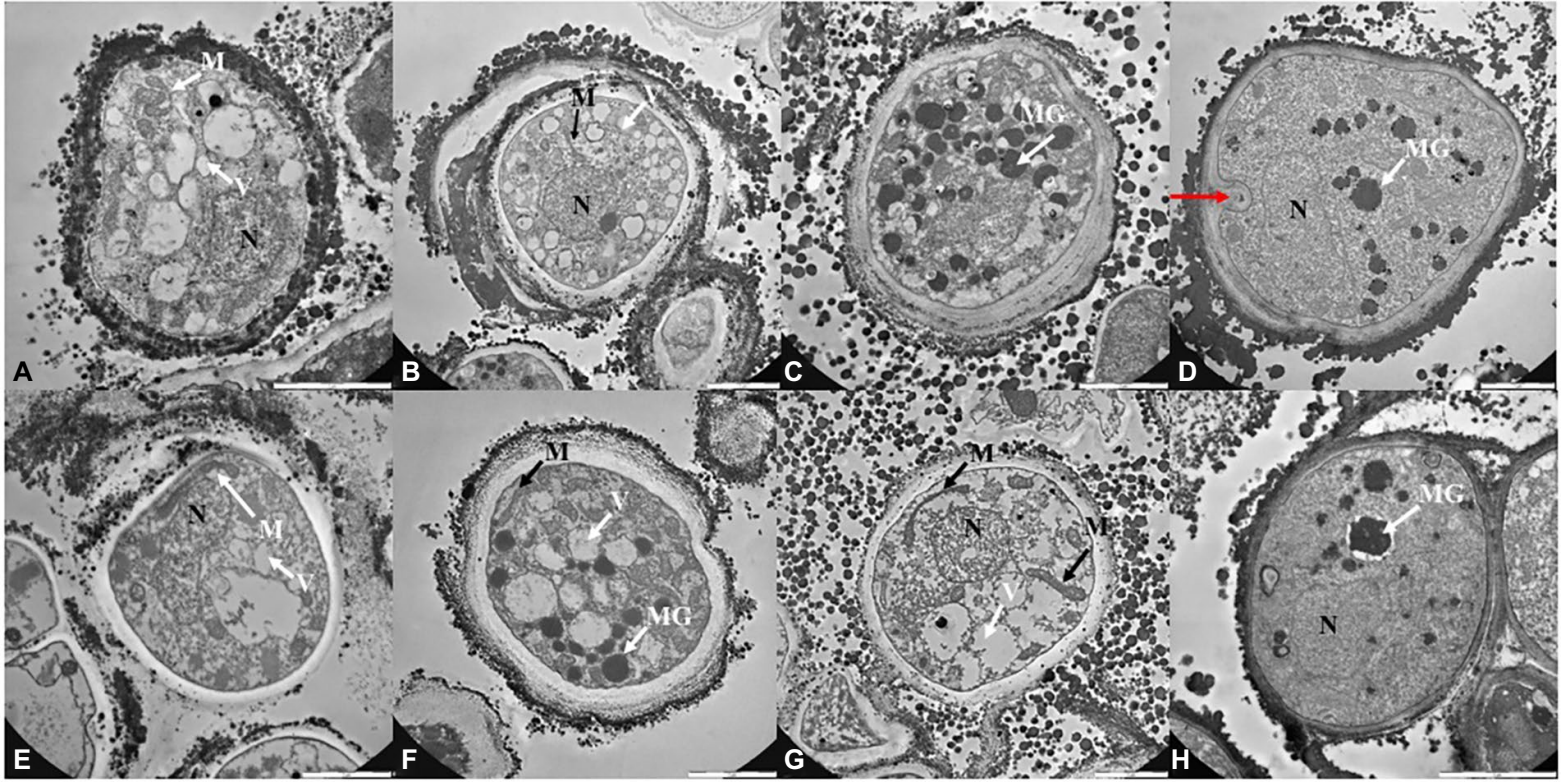
TEM images of *C. antarcticus* cells grown on **(A)** MEA (no salt, control), **(B)** 50 mM, **(C)** 120 mM and **(D)** 145 mM of Mg-perchlorate; **(E)** MEA (no salt, control), **(F)** 50 mM, **(G)** 150 mM, and **(H)** 200 mM of Ca-perchlorate. Scale bar: 2 μm. N, nucleus; M, mitochodrion; DM, damaged mitochodrion; CW, cell wall; LB, lipid body; V, vacuoles; MG, melanin granules; red arrow, cell wall indentation.

TEM observations (Figures 4, 5; Supplementary Figure S3) were performed only on samples grown at the lower, the intermediated and the higher perchlorate concentration tested, as for the metabolic activity assay. Control samples (0 mM, Figures 4A,E) showed a well-organized cell with dense cytoplasm and well-visible cellular structures. Nucleus, mitochondrions (Figures 4A,E, white arrows, M) and vacuoles are highly preserved (Figures 4A,E, white arrows, V). Lipid bodies are also visible (Figure 4A, white arrow, LB). A well-preserved cell-membranes and cell-walls have been reported (Figures 4A,E, black arrows). Fungal colonies grown on Na-perchlorate medium (50, 150 and 220 mM, Figures 4B–D; respectively) showed a well-preserved cell-membranes and cell walls (Figures 4B–D, black arrows, CW). The internal structures are scarcely distinguishable: vacuoles and lipid bodies are still visible unlike the nuclei and mitochondrions (Figures 4C,D, black arrows, V and white arrows, LB). Melanin granules are reported in Figure 4C (white arrow, MG). Figures 4F–H reported TEM images of cells grown on increasing K-perchlorate concentrations. Nuclei (N) are well visible in K-perchlorate of 50- and 80-mM concentrations (Figures 4F,G) Structures indicated with a black arrows correspond to damaged mitochondria (DM) (Figures 4F,G). In Figure 4H, peculiar vacuolar structures surrounded by melanin granules are reported (red arrows). Figures 5B–D shows TEM images of fungal cells grown on Mg-perchlorate medium. Control sample (0 mM) are reported in Figure 5A and showed well-preserved nucleus, mitochondria and vacuoles. Figure 5B reported well-preserved cells grown on 50 mM of Mg-perchlorate with nucleus, mitochondrion (black arrow) and vacuole (white arrow). On the contrary, cells grown at higher concentrations (120 and 145 mM, Figures 5C,D; respectively) showed a not well-organized cytoplasm, with a high amount of melanin granules (white arrows). Cells at 145 mM of Mg-perchlorate showed also a cell wall indentation (Figure 5D, red arrow), indicating the start of cell death process.

Similarly to control sample (0 mM, Figure 5E), well-structured cells were reported for samples grown at 50, 150 mM and 200 of Ca-perchlorate (Figures 5F–H). There is an increase in vacuoles size (Figures 5F,G, white arrows), while nuclei and mitochondria are still distinguishable (Figures 5F,G, black and white arrows). A slack cytoplasm characterized by abundant melanin granules was reported for cells grown on 200 mM of Ca-perchlorate (Figures 5F,H).

## Discussions

The aim of this work was to investigate the survivability, metabolic activity recovery, cell- membrane damages and the ultrastructural damages of the Antarctic cryptoendolithic black fungus *C. antarcticus* after grown in presence of perchlorates at increasing concentrations. The fungus was also tested in the presence of Mars-relevant perchlorate concentration, as 2.4 mM perchlorate ions (0.4 wt% of Mg(ClO_4_)_2_ and 0.6 wt% of Ca(ClO_4_)_2_), as reported by the NASA’s Phoenix Mars Lander (Hecht et al., 2009). The results might give insight for the concept of habitability beyond Earth, in particular on Mars where perchlorates are abundant.

Perchlorate ions are known to damage the main functions of terrestrial organisms; they interrupt a series of metabolic processes, and also act as oxidizing agents causing cell-membrane damages (Urbansky, 1998). On Earth, the occurrence of perchlorates is limited only in certain arid environments at concentrations ranging from 10^−1^ to 10^6^ μg/kg (Trumpolt et al., 2005; Catling et al., 2010; Jackson et al., 2015, 2016); while on Mars they have been detected in good concentrations at different location, up to 10^7^ μg/kg (Hecht et al., 2009).

In addition, their importance is given by the fact that they favor the presence of transient liquid water on the Martian surface (one of the main factors to determine the habitability of a planet). Although liquid water is not a stable condition, geomorphological structures named Recurring Slope Lineae (RSL), have been detected on the planet in evidence of the seasonal presence of water (McEwen et al., 2014). Although the prevalent theory is that these structures are possibly connected to liquid water, alternative theories associate them to dry granular fluxes and seasonal variations in the amount of near-surface water adsorbed (McEwen et al., 2011).

Perchlorates, in brines in the near surface, are able to maintain fluid state for several days under Martian environment conditions (Chevrier et al., 2009; Hennings et al., 2013; Orosei et al., 2018). The presence of perchlorates in Martian regolith may reduce the crystallization temperature of water, also allowing the activity of putative microorganisms on Mars (Beblo-Vranesevic et al., 2020), and persisting in the first layer of regolith could support the preservation of cellular components (Primm et al., 2017).

In this context, the exposure of *C. antarcticus* cells to increasing perchlorate concentrations revealed that the survival was (i) not affected in 80 mM of K-perchlorate and 80 mM of Mg-perchlorate; (ii) halved at 90 mM of K-perchlorate and 5 mM of Ca-perchlorate; and (iii) completely inhibited in 230 mM of Na- perchlorate, 150 mM of Mg-perchlorate and 210 mM of Ca-perchlorates (Figure 1).

We are not able to test the limit of survival in K-perchlorate due to its solubility limit. In addition, an excellent survivability, around 80% of survivors, was found for cultivation medium containing the perchlorates concentration finding at the Phoenix landing site (2.4 mM, Supplementary Figure S1). The survival results are in accordance with the cell-membrane integrity evaluation. Indeed, a decrease of cells with undamaged cell-membranes as the perchlorate concentration increase was reported (Figure 3). These results are in accordance the analyses of the ultrastructure analyses, which showed cells with damaged cell-membranes as a consequence of the presence of perchlorates (Supplementary Figure S3). According to other studies, certain anions, such as perchlorate, can also cause chaotropic stress, which can lead macromolecules to become unstable (Ball and Hallsworth, 2015). For example, it has been demonstrated that *Pseudomonas putida* responds to chaotropic solute-induced water stress by upregulating proteins that are essential for the stability of biological macromolecules and cellular membrane structure (Heinz et al., 2022).

It has been hypothesized that meristematic grow, a peculiarity of black yeast where *C. antarcticus* belongs, could enhance the ability to withstand in presence of perchlorates (Wollenzien et al., 1995).

Similar concentrations were reported for the survivability of the cyanobacterium *Chroococcidiopsis.*

CCMEE 029 (Billi et al., 2021): a long-term exposure did not affect the survivability of the cyanobacterium up to 5 mM Na-, Mg- and Ca-perchlorates. Accordingly to our results, a decrease with the increase of perchlorate concentration was reported: a reduction was observed in 50 mM of Na-perchlorate and a complete loss was reported at 100 mM of Ca- and Mg-perchlorates (Billi et al., 2021). At the concentration of 100 mM of Na-perchlorate, the survivability of *C. antarcticus* is reduced up to 23% of survivors (Figure 1A). Compared to the halotolerant yeast *D. hansenii,* which is able to grow up to 2.4 M of Na-perchlorate (Heinz et al., 2020), *C. antarcticus* is able to grow at maximum 0.22 M of Na-perchlorate (with around 1% of survivors, Figure 1A); this result is still noteworthy considering that *C. antarcticus* is not a halotolerant specie. Several other studies reported the growth of non-fungal species in perchlorate solutions. The halophilic archaea *Halobacterium* strain NRC-1, *Hbt. salinarum* R1, *Haloferax volcanii*, *Hfx. mediterranei, Hfx. denitrificans, Hfx. gibbonsii, Haloarcula marismortui, and Har. vallismortis* demonstrated their ability to grow up to 0.4 M of Na-perchlorate (Oren et al., 2014). Al Soudi et al. (2017), recorded a robust grow of *Halomonas venusta* at 1 M of Na-perchlorate, while *Planococcus halocryophilus* survived up to 1.1 M of Na-perchlorate (Heinz et al., 2019). *Halorubrum lacusprofundi* showed a higher sensitivity to Mg-perchlorate than Na-perchlorate (50% inhibition at 0.3 M Na-perchlorate versus 0.1 M for Mg-perchlorate; Laye and DasSarma, 2018), while no growth was reported for *Chroococcus cf. membraninus*, *Mycrocystis aeruginosa*, *Calochaete cimrmanii*, *Kastovskya adunca* cyanobacteria species in presence of the same compound (Rzymski et al., 2022). *C. thermalis* was the most tolerant among different tested strain of cyanobacteria, maintaining growth throughout the 0.25–1.0% magnesium perchlorate concentration range (Rzymski et al., 2022).

Surprisingly, the tolerant bacterium *Hydrogenothermus marinus* resisted to an exposure of 5 min in 5 M of Na-perchlorates at room temperature, reported not change in survivability, while a 96 h-exposure resulted in a reduced survivability (Beblo-Vranesevic et al., 2017). Even if these studies demonstrate the survival of different bacterial and fungal strains on perchlorates expanding the limits of life and the habitability of Mars, the presence of irradiation, associated with perchlorates, should be taken into account since is one of the main limiting factors occurring on the planet. Indeed, the combined exposure of *Bacillus subtilis* to the 0.6 wt% of Mg-perchlorate and to monochromatic UV irradiation (200 and 315 nm), showed a complete loss of vitality after 30 s after the exposure to radiation (Wadsworth and Cockell, 2017).

The water activity is known to be a major limiting factor for growth (Stevenson et al., 2015). Since the addition of salts to water may decrease the water activity, this parameter was determined for each tested concentration. All the measurements are in the range between 0.92 and 0.94 (Table 2). The limiting value of water activity to support life is ≈0.6 (Stevenson et al., 2015). The survival results were further confirmed by the metabolic activity recovery analyses. A decrease in metabolic activity with the increasing of perchlorate concentration was reported in all samples (Figure 2).

Changes at ultrastructural level were detected with TEM observations, highlighting the stress responses to perchlorates. In fact, TEM images highlighted a slack cytoplasm in the cells grown on perchlorates, comparing with the dense cytoplasm of control cells (0 mM). In control and low-perchlorate concentration, nuclei, mitochondria, vacuoles and lipid bodies are generally well-preserved. Cells grown on Na-perchlorate (Figures 4B–D), still showed a cytoplasm with vacuoles and lipid bodies. At the concentration of 220 mM of Na-perchlorate, a decrease in cell-wall thickness was observed (Figure 4D). This result is directly compared to the survival since we reported only 0.3% of the survivors in the samples where a reduction of cell-wall thickness is observed (Figure 4D). Similar results were obtained at the concentration of 150 and 200 mM of Ca-perchlorate (where we found 21 and 0.2% of the survivors, respectively) (Figures 5G,H). The good value of metabolic activity (0.75, 0.9, 0.7% of metabolic activity of cells grown on 220 mM of Na-perchlorate, 150 and 200 mM of Ca-perchlorate, respectively, Figure 2) can be explained assuming that the few alive cells are actively trying to repair the damages induced by salts. Cell-wall modifications are probably related to changes in the molecular composition: a branching degree of the polysaccharides, an elevated β-1,3-glucan level (Lesage and Bussey, 2006), or the incorporation of proteins into the cell wall as a result of changed glucan synthesis (Smits et al., 2001), or elevated chitin levels (Pessoni et al., 2005). Colonies grown on K-perchlorate (Figures 4F–H), showed the presence of damaged mitochondria, compared to control sample (0 mM, Figure 4E) and, at higher concentration, vacuolar structures, surrounded by melanin granules, are reported (Figure 4H, red arrows). In samples grown on Mg- and Ca-perchlorates (Figures 5B–D,F), lipid bodies and melanin granules are accumulated within the cytoplasm. It is demonstrated that pigmentation may protect cells from various stressors (Chung et al., 2003; Mandal et al., 2007). In this context, cells may act a protection strategy accumulating and secreting melanin granules. Similar structures were reported in TEM images of *H. werneckii* after growth on NaCl (Kogej et al., 2007). Melanin granules may limit the cell-wall permeability by reducing the size of cell wall pores. In fact, a reduction pores size from 10.6 in non-melanized to 4 nm in melanized cell of *Cryptococcus neoformans*, was reported (Jacobson and Ikeda, 2005). To better understand the mechanism, a comparison with non-melanized *C. antarcticus* cells could be performed.

Recently, it has been demonstrated that osmolytes production increases after simulated space stressors, including dehydration and vacuum (Gevi et al., 2022). Also, other studies demonstrated a predominant production of glycerol as the most abundant osmolytes in *Aspergillus wentii* after growing on salt media (Danilova et al., 2022). This is also confirmed by Heinz et al. (2022) in which a proteomic approach demonstrated that *D. hansenii* is able to produce glycerol as compatible solute and antagonizes osmotic.

As reported in Kogej et al. (2007), a combination of compatible solutes supported by a cell-wall melanization, was useful for the growth of *H. werneckii* on NaCl concentrations. Several studies also reported that the accumulation of pigments (e.g., carotenoids) may mitigate oxidative stress, as highlighted for *Chroococcidiopsis* sp. after the exposure to gamma rays (Baqué et al., 2020) and for *C. thermalis* and *L. foveolarum* under perchlorate exposure (Rzymski et al., 2022).

In overall, the survivability of the fungus *C. antarcticus* to increased salinities are similar to that of halotolerant yeast species. The current work demostrated that a non-halophilic terrestrial extremophilic fungus can growth in perchlorates media, including Mars relevant perchlorate salts. This is particularly important in the context of the habitability of Solar System, considering that perchlorate salts have been found on Mars, and potentially near the oceans of icy worlds, as Europa and Enceladus (since it has been demonstrated that Enceladus’ water plumes contain dissolved salts; Postberg et al., 2009; Waite et al., 2017). Further studies aiming at investigate the mechanisms at the basis of the fungal perchlorate tolerance, trying to identify the genes involved in the resistance, are necessary.

The identification of novel genes involved in the molecular strategies allowing microorganism to survive perchlorate conditions could also be exploited to create perchlorate-resistance strains to develop Bioregenerative Life Support Systems (BLSS) based on *in situ* resource utilization (ISRU) (e.g., to remove perchlorate in Martian soil). A necessary further step will be the investigation of the combined effects of perchlorates and radiation (e.g., UV radiation) to better understand if conditions similar to those experienced on Martian surface might allow or not the survival of putative Earth-like microorganisms, and this has also implication for the planetary protection.

## Conclusion

The effects of perchlorates prevalent on the surface of Mars are of significant interest to astrobiology from the perspective of the life searching. Perchlorates have been detected at many locations and at different concentrations on Mars surface. The ubiquity presence of perchlorate salts on the planet could suggest the possibility of transient liquid water on the surface, thanks to the deliquescence of the salts. This study provide, for the first time, insight about the resistance of the black fungus *C. antarcticus* to four perchlorate species, demonstrating to survive at 0.4–0.6 wt% of Mg(ClO_4_)_2_ and Ca(ClO_4_)_2,_ and up to 220 mM of Na-, 200 mM of Ca-, 145 mM of Mg- and 90 mM of K-perchlorates, with implication on habitability in planetary context.

## Data availability statement

The raw data supporting the conclusions of this article will be made available by the authors, without undue reservation.

## Author contributions

AC, CP, and SO designed the research. AC performed the experiments. AC and CP analyzed data. AC drafted the paper with inputs from all other authors. All authors contributed to the article and approved the submitted version.

## Funding

This work was supported by the Italian Space Agency by ASI grant (ASI N. 2019-3-U.0, Life in Space). The Italian National Program of Antarctic Researches (PNRA) and the Italian National Antarctic Museum “Felice Ippolito” (MNA) are also acknowledged for funding the collection of Antarctic samples CCFEE.

## Conflict of interest

The authors declare that the research was conducted in the absence of any commercial or financial relationships that could be construed as a potential conflict of interest.

## Publisher’s note

All claims expressed in this article are solely those of the authors and do not necessarily represent those of their affiliated organizations, or those of the publisher, the editors and the reviewers. Any product that may be evaluated in this article, or claim that may be made by its manufacturer, is not guaranteed or endorsed by the publisher.
