# Supplementary material for: Survival, metabolic activity, and ultrastructural damages of Antarctic black fungus in perchlorates media
Source: Front Microbiol. 2022 Nov 29;13:992077. doi: 10.3389/fmicb.2022.992077 (PMC9744811; doi:10.3389/fmicb.2022.992077)
Supplement: Supplementary file 1 [file Data_Sheet_1.docx]

**Survival ability, metabolic activity recovery and ultrastructural damages of Antarctic black fungus in perchlorates media**

Cassaro A.^1^, Pacelli C.^1,2,*^, Onofri S.^1^

^1^Department of Ecological and Biological Sciences, University of Tuscia, Largo dell’Università snc, Viterbo, Italy

^2^ Human Spaceflight and Scientific Research Unit, Italian Space Agency, via del Politecnico, Rome, Italy

**Supplementary Material**

**Growth tolerance to Mars-relevant perchlorate salts**

**
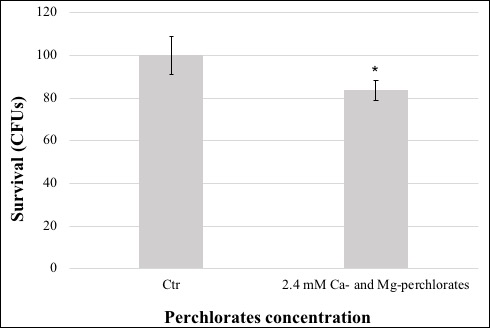
**

**Figure S1.** Survival ability of *C. antarcticus* colonies after growth on 2.4 mM (0.4 wt% of Mg(ClO_4_)_2_ and 0.6 wt% of Ca(ClO_4_)_2_) cultivation medium. Significant differences were calculated by *t test* with * = *p* < 0.05 and ** = *p* < 0.001.


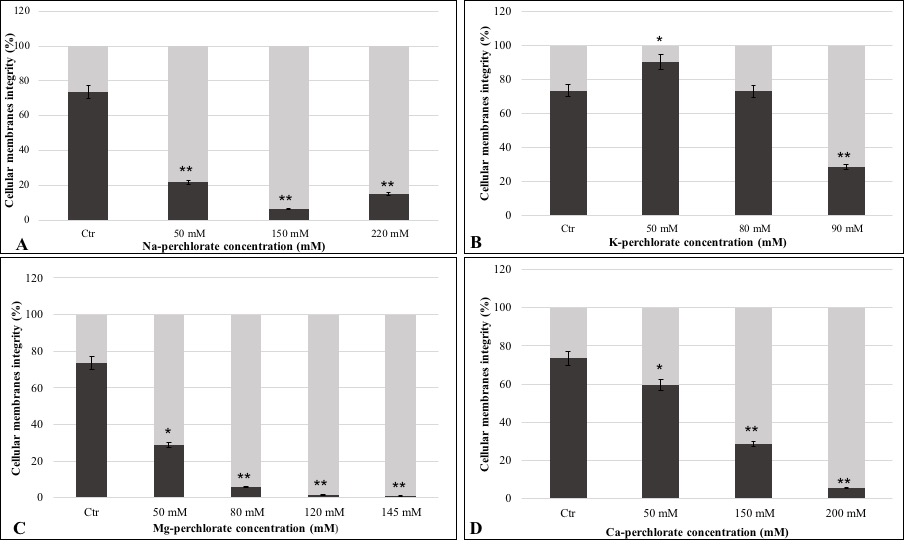
**Cellular membranes integrity assessment (PMA assay)**

**Figure S2.** Percentage of undamaged and damaged cellular membranes measured with PMA assay coupled with qPCR of *C. antarcticus* colonies grown on different perchlorates concentrations. **A**) Na-, **B**) K-, **C**) Mg-, and **D**) Ca-perchlorates. All concentrations are expressed in mM. Significant differences were calculated by *t test* with * = *p* < 0.05 and ** = *p* < 0.001.

**Ultrastructural investigation: TEM observations**

**
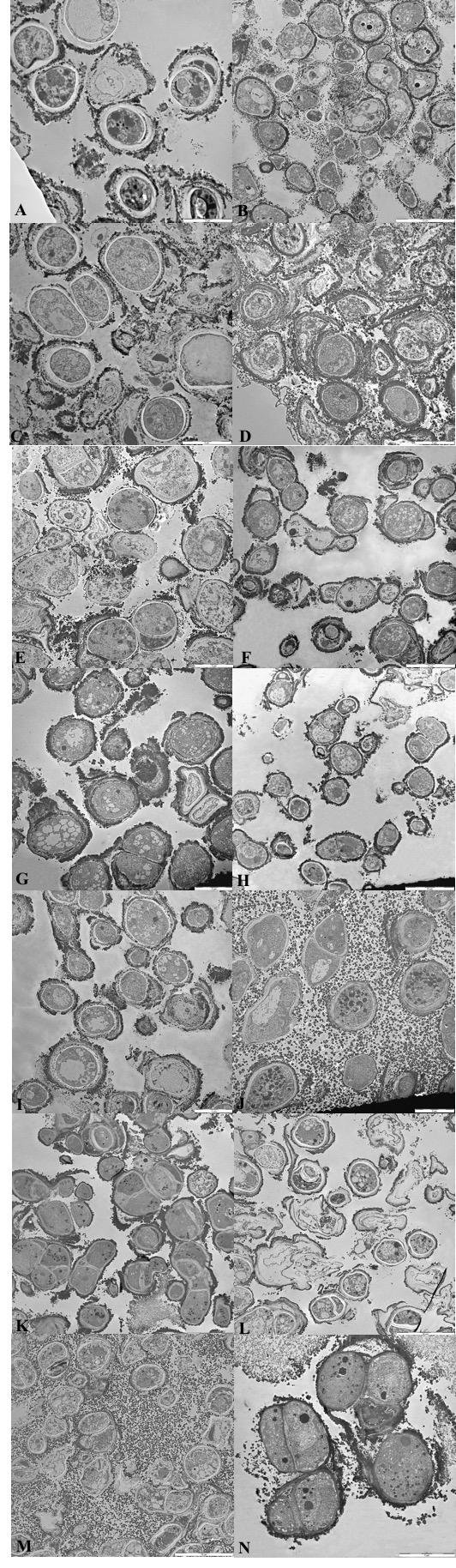
**

**Figure S3.** TEM images of *C. antarcticus* cells grown on A, B) MEA (no salts, controls), C) 50 mM, D) 150 mM and E) 220 mM of Na-perchlorate; F) 50 mM, G) 80 mM and H) 90 mM of K-perchlorate; I) 50 mM, J) 120 mM and K) 145 mM of Mg-perchlorate; L) 50 mM, M) 150 mM and N) 200 mM of Ca-perchlorate.
